# Health Literacy Is Associated with Activities of Daily Living of Patients Participating in Cardiac Rehabilitation: A Multicenter Clinical Study

**DOI:** 10.3390/ijerph192416550

**Published:** 2022-12-09

**Authors:** Yuji Kanejima, Kazuhiro P. Izawa, Masahiro Kitamura, Kodai Ishihara, Asami Ogura, Ikko Kubo, Hitomi Nagashima, Hideto Tawa, Daisuke Matsumoto, Ikki Shimizu

**Affiliations:** 1 Department of Public Health, Graduate School of Health Sciences, Kobe University, Kobe 654-0142, Japan; 2 Cardiovascular Stroke Renal Project (CRP), Kobe 654-0142, Japan; 3 Department of Rehabilitation, Kobe City Medical Center General Hospital, Kobe 650-0047, Japan; 4 Department of Physical Therapy, Faculty of Rehabilitation, Reiwa Health Sciences University, Fukuoka 811-0213, Japan; 5 Department of Rehabilitation, Sakakibara Heart Institute of Okayama, Okayama 700-0804, Japan; 6 Department of Rehabilitation, Sanda City Hospital, Sanda 669-1311, Japan; 7 Department of Rehabilitation, Yodogawa Christian Hospital, Osaka 533-0024, Japan; 8 Department of Rehabilitation, Shinyukuhashi Hospital, Yukuhashi 824-0026, Japan; 9 Department of Cardiology, Sanda City Hospital, Sanda 669-1311, Japan; 10 Department of Cardiovascular Medicine, Yodogawa Christian Hospital, Osaka 533-0024, Japan; 11 Department of Diabetes, Sakakibara Heart Institute of Okayama, Okayama 700-0804, Japan

**Keywords:** cardiac rehabilitation, health literacy, activities of daily living, multicenter clinical study

## Abstract

The activities of daily living (ADL) in patients with cardiac disease tend to decline. A previous study revealed that ADL relates to physical and cognitive functions associated with health literacy (HL). However, the relationship between HL and ADL is not well documented. This study aimed to clarify this relationship among patients participating in cardiac rehabilitation. This multicenter study, the Kobe-Cardiac Rehabilitation project for people around the World (K-CREW), included patients who participated in cardiac rehabilitation from October 2020 to December 2021. Patients with probable dementia and difficulty walking alone were excluded. We used the 14-item Health Literacy Scale (HLS-14) to assess HL and the Functional Independence Measure (FIM) to assess ADL at discharge. Patients were divided by their HLS-14 score into the low HL group (<50 points) or the high HL group (≥50 points). We analyzed the relationship between the HLS-14 and FIM scores. We investigated 268 cardiac rehabilitation patients (median age, 71.0 years; male ratio, 76.9%). Low HL patients accounted for 51.1% of all patients and had significantly lower motor and cognitive FIM scores. Functional HL related better to the FIM scores (r = 0.28–0.36) than did other HL subclasses. Multiple regression analysis identified HLS-14 as an explanatory variable (*p* = 0.002) for the total FIM score. Patients with low HL had significantly lower ADL than those with high HL. These findings underscore the importance of considering HL in cardiac rehabilitation.

## 1. Introduction

Health literacy (HL) is considered “the cognitive and social skills which determine the motivation and ability of individuals to gain access to, understand and use information in ways which promote and maintain health” [1]. Patients with low HL tend to have difficulties with complex health tasks and abilities, such as understanding health information, having limited access to health care, and expressing their concerns, emotions, and needs [2]. Low HL is related to an unhealthy lifestyle and risks of cardiac disease [2]. Moreover, patients with cardiac disease and low HL tend to have a poorer health-related quality of life (QOL), physical function, and higher rates of mortality and readmission [2,3]. HL has attracted attention for its importance in increasing prognosis and QOL.

Cardiac rehabilitation is composed of exercise and education programs that improve exercise capacity, QOL, and prognoses related to rates of mortality and readmission [4]. Not only exercise programs but also education programs are essential. Comprehensive cardiac rehabilitation programs that included patient education reduced mortality by 73% compared to exercise-only programs [4]. In addition, HL can predict gains in cardiac knowledge from a patient education program in cardiac rehabilitation [5]. Therefore, HL is necessary for cardiac rehabilitation, especially for patients with low HL, so that they can better understand and apply the contents of patient education.

The activities of daily living (ADL) are the fundamental skills required to care for oneself independently, such as eating, grooming, dressing, toileting, transferring, and ambulating [6]. Patients requiring ADL support have increased healthcare costs and higher rates of mortality and readmission than independent patients [6,7]. Assessment of ADL is vital in routine patient assessment and assists medical staff in assessing the patient’s status and in planning and intervening appropriately. Assessment of functionality in ADL should become standard practice for all patients as it can affect people of all ages [8]. Improving ADL may lead to a better prognosis and QOL.

HL is related to physical and cognitive function in cardiac disease patients [2,3], but physical and cognitive functions in patients with cardiac disease are decreased, which can cause ADL dependence. Physical and cognitive functions are common factors between HL and ADL [9,10]. A previous study showed that limited HL was related to ADL dependence in community-dwelling older adults [11]. For example, limited HL can cause problems with preventive services, delayed diagnoses, and adherence to medical instructions [11]. However, there are few reports on the relationship between HL and ADL in patients with cardiac disease participating in cardiac rehabilitation. So we hypothesized that patients participating in cardiac rehabilitation with low HL would have poorer ADL than patients with high HL. In addition, the present study aimed to clarify the relationship between HL and ADL in patients participating in cardiac rehabilitation and to consider further practical interventions for cardiac rehabilitation.

## 2. Materials and Methods

### 2.1. Study Design and Eligibility Criteria

The present study was a multicenter clinical study named K-CREW (Kobe-Cardiac Rehabilitation project for people around the World). K-CREW includes four affiliated small-to-medium-scale hospitals with 200–580 beds as follows: Sakakibara Heart Institute of Okayama, Sanda City Hospital, Shinyukuhashi Hospital, and Yodogawa Christian Hospital, all of which conduct cardiac rehabilitation. The advantages of a multicenter clinical trial are to obtain a more heterogeneous sampling of subjects and to bring generalizability to the investigation [12]. The inclusion criteria were defined as follows: patients who (1) were admitted to affiliated hospitals from 1 October 2020 to 31 December 2021, (2) participated in cardiac rehabilitation, (3) were hospitalized for more than five days, and (4) had not been hospitalized for a medical check-up (e.g., admission for coronary angiography (CAG)), percutaneous coronary intervention (PCI) without rehabilitation, ablation, or pacemaker battery replacement. After including patients who met these criteria, the present study excluded patients with probable dementia (based on diagnosis or Mini-Mental State Examination (MMSE) < 24), difficulty walking alone, disagreement with informed consent, hospital death, and data deficits. Patients were divided into seven disease groups according to the primary diagnosis: (1) heart failure, (2) myocardial infarction, (3) angina pectoris, (4) aortic dissection, (5) aortic aneurysm, (6) valve diseases, and (7) other diseases. The co-authors provided informed consent to patients who met the eligibility criteria during hospitalization and asked them to participate in the required exercise and assessment of cardiac rehabilitation. We describe the details of the exercise and assessment below.

### 2.2. Phase 1 Cardiac Rehabilitation

Affiliated hospitals conducted cardiac rehabilitation based on the Japanese Circulation Society guidelines for rehabilitation in patients with cardiovascular disease [4]. Phase 1 cardiac rehabilitation started within three days after admission and cardiac surgery [13]. The exercise program was composed of aerobic exercise and resistance training for 5–7 days a week. Aerobic exercise for less than 25 min a day and resistance training for 10–20 min were conducted by adjusting them to the patient’s condition. The exercise intensity was coordinated to a Borg scale value of 11–13 or just under the patient’s threshold of anaerobic metabolism. Patient education consisted of lectures about diseases, lifestyle, nutrition, medications, and exercise provided by a doctor, nurse, registered dietitian, pharmacist, and physical or occupational therapist, respectively. The present study did not consider the patient’s level of HL in regard to the education programs.

### 2.3. Patient Characteristics

We collected the following data on patient characteristics: age, sex, body mass index (BMI), education, employment, living together, smoking, marriage status, levels of serum hemoglobin, creatinine, and brain natriuretic peptide (BNP), estimated glomerular filtration rate (eGFR), Geriatric Nutritional Risk Index (GNRI) [14], comorbidities, Charlson Comorbidity Index [15], and left ventricular ejection fraction (EF) at the time of admission. The GNRI values were classified as follows: <82: major, 82 to <92: moderate, 92 to <98: low, and ≥98: no nutrition-related risk. [14] We also assessed cognitive function with the MMSE and the Japanese version of the Montreal Cognitive Assessment (MoCA-J) and checked prescriptions at discharge.

### 2.4. Assessment of Health Literacy

We used the 14-item Health Literacy Scale (HLS-14) to assess the patient’s HL [16]. The HLS-14 has 14 questions, with each question ranging from one to five points, for a total score of 14 to 70 points. This assessment consisted of three subclasses of HL: functional, communicative, and critical. Functional HL means sufficient basic skills in reading and writing in daily situations; communicative HL means skills to participate in daily activities and extract information on one’s own; critical HL means more skills to critically analyze information and use the information to better control the situation. The original study showed high reliability and validity (Cronbach’s α: 0.81, CFI: 0.912, NFI: 0.905, and RMSEA: 0.082) [16]. The present study divided the patients into two groups according to a cutoff of 50 points on the HLS-14, as in the original study (low HL group: <50 points, high HL group: ≥50 points) [16].

### 2.5. Outcome

The primary outcome was the Functional Independence Measure (FIM) as an assessment tool for ADL [17,18]. The FIM consists of 18 items ranging from one to seven points, which were scored by the level of dependence in actual ADL. A higher FIM score means greater independence in ADL. The FIM is divided into the motor and cognitive domains. The motor FIM has 13 items, including self-care, sphincter control, transfers, and locomotion, and the cognitive FIM has five items, including communication and social cognition. One researcher collected the data on patient characteristics from their medical records at admission, and one to three cardiac rehabilitation staff members assessed each patient’s FIM and asked patients to answer the HLS-14 and cognitive function questionnaire (Mini Mental State Examination and Japanese version of Montreal Cognitive Assessment) at discharge.

### 2.6. Statistical Analysis

All analyses were conducted on patients with no missing data for any of the variables. First, we compared the low HL and high HL groups using the Wilcoxon rank-sum test for continuous variables and Pearson’s Chi-squared test or Fisher’s exact test for categorical variables. Next, Spearman’s rank correlation coefficients were calculated in each subclass between the HL (functional, communicative, critical, and total scores) and FIM (motor, cognitive, and total scores) scores. We regarded the strength of correlation as follows: r = 0.00–0.10: negligible; 0.10–0.39: weak; 0.40–0.69: moderate; 0.70–0.89: strong; and 0.90–1.00: very strong correlation. [19] Finally, a multiple linear regression analysis was conducted for the total FIM score as a dependent variable. We selected independent variables related to HL or ADL according to previous studies: age, sex, BMI, primary diagnosis of heart failure or myocardial infarction, mild cognitive impairment (MCI), stroke, renal disease, EF, GNRI, smoking, education, occupation, marriage, living together, and HLS-14 [20,21,22]. Univariable and multivariable models were constructed. 

We used R studio (ver. 1.3.959) as analysis software [23] and regarded the significance level as *p* < 0.05. The ethics board of Kobe University approved the present study on 12 August 2020 (No. 951), and each affiliated hospital received approval from its local ethics committee. The study procedures were carried out in accordance with the Declaration of Helsinki [24].

## 3. Results

### 3.1. Patient Characteristics

**Figure 1:**
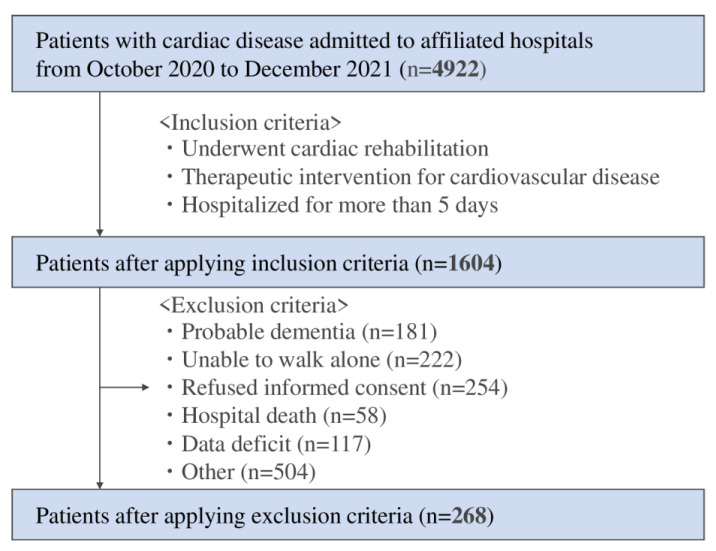
Flow chart of the study participants.

Of the 4992 patients with cardiac disease admitted to the affiliated hospitals during the study period, 1604 patients met the inclusion criteria, including being hospitalized for more than five days and undergoing cardiac rehabilitation. Thereafter, patients admitted for CAG (*n* = 1066), PCI without rehabilitation (*n* = 1272), ablation (*n* = 181), and pacemaker battery exchange (*n* = 24) were excluded due to short-term admission. After further excluding patients who could not walk alone or had dementia, 268 patients were finally included in the present study (Figure 1).

The patients’ median age was 71.0 years, and the ratio of males was 76.9%. The frequency of the primary diagnosis of each disease was as follows: heart failure: 35.8%; myocardial infarction: 42.2%; angina pectoris: 9.7%; aortic dissection: 1.1%; aortic aneurysm: 1.5%; valve diseases: 6.0%; and other diseases: 4.1%. Other diseases included infective endocarditis, ischemic cardiomyopathy, spontaneous coronary artery dissection, atrial septal defect, atrial fibrillation, post-cardiopulmonary arrest, and constrictive pericarditis. The overall patient median scores were motor FIM: 90.0 points, cognitive FIM: 35.0 points, and total FIM: 125.0 points. The median HLS-14 scores were functional HL: 21.0 points, communicative HL: 16.0 points, critical HL: 12.0 points, and total HL: 49.0 points.

### 3.2. Comparison between Low HL Group and High HL Group

The low HL group comprised 51.1% of the patients and varied from 40.7–59.6% by affiliated hospitals. The median total HLS-14 score was 43.0 points in the low HL group and 54.0 points in the high HL group. Patients in the low HL group tended to be admitted more for heart failure, whereas those in the high HL group tended to be admitted for myocardial infarction. Significant differences were observed in dyslipidemia, BNP, statin, and each domain of the HLS-14 scores as a result of comparative analysis. The motor, cognitive, and total FIM scores were significantly lower in the low HL group compared to the high HL group. Concretely, the median (IQR) scores for motor FIM were low HL 90.0 (88.0–91.0) vs. high HL 91.0 (89.0–91.0) points, for cognitive FIM were low HL 35.0 (33.0–35.0) vs. high HL 35.0 (35.0–35.0) points, and for total FIM were low HL 124.0 (120.0–126.0) vs. high HL 125.0 (124.0–126.0) points (Table 1).

### 3.3. Correlation Coefficient

**Figure 2:**
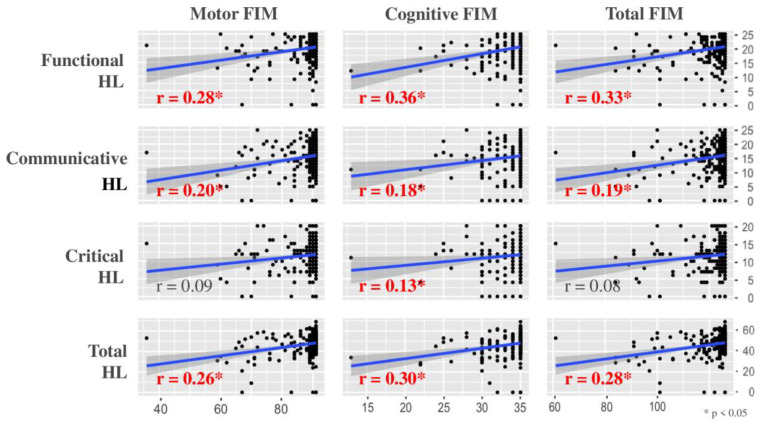
Correlation between health literacy (HL) and the Functional Independence Measure (FIM).

Figure 2 summarizes the correlation coefficient of each subclass between HL and the FIM. Communicative and total HL showed a significantly positive correlation with the FIM, and functional HL correlated most highly with the FIM. However, each correlation coefficient between the HL and FIM scores was less than 0.40, which indicates a weak correlation.

### 3.4. Multiple Linear Regression Analysis

All variables, except living together, smoking, and EF, were significantly associated with the total FIM score in the univariate analysis. The multiple linear regression analysis identified the factors significantly associated with the total FIM score in terms of age, primary diagnosis for heart failure, MCI, and stroke as comorbidities, marriage, living together, and the HLS-14. The adjusted R2 value was 0.40, and the F-statistic was 12.08 on the 16 variables in this analysis model (Table 2).

## 4. Discussion

### 4.1. Summary

The results of the present study show that patients with low HL participating in cardiac rehabilitation had significantly lower motor, cognitive, and total FIM scores than patients with high HL and that functional HL correlated most highly with the FIM in the subclasses of the HLS-14. Multiple regression analysis revealed that the HLS-14 remained an explanatory variable for the total FIM score.

### 4.2. Comparison with Previous Studies

HL relates to physical and cognitive functions in cardiac disease patients [2,25]. Patients in the low HL group in the present study had lower physical and cognitive FIM scores than those in the high HL group, which supports the results of previous studies [2,25]. In addition, a previous study using a questionnaire to assess ADL showed that HL and ADL were related. [11] However, the present study assessed ADL and found that patients actually performed during hospitalization. That we analyzed a clinically realistic situation can be regarded as the strong point of the present study.

In a Taiwanese study focusing on ADL in patients with cardiac disease [26], the median age (70 years), male ratio (60%), and length of stay (17 days) were similar to those of the present study. Still, this comparative study included more patients who had undergone cardiac surgery (52%) and fewer patients who were discharged home (76%). The total score of the FIM at discharge in the present study was higher than that in the comparative study (median: 125.0 vs. 113.0 points), indicating that patients in the present study tended to be more independent. The cutoff value of the motor FIM is 63.0 points for discharge to home and 74.5 points for 90-day readmission rates [27]. The median motor FIM was 90.0 points, which shows that the present study supports the results of the previous study, and patients may have a good prognosis, such as lower rates of 90-day readmission.

The minimum clinically important differences (MCID) for the total FIM score are 22.0 points, 17.0 points for motor FIM, and 3 points for cognitive FIM [28]. There was no MCID between the low HL group and the high HL group because many patients scored almost the full score on the FIM. A previous study revealed that the FIM has a ceiling effect [29]. The results of the present analysis affected this feature of the FIM. In addition, we excluded patients who could not walk alone as an indication of impaired motor function and those who probably had dementia as an indication of impaired cognitive function. In other words, we might have excluded patients with poor ADL and low levels of independence, and we thought that this fact likely affected the analysis results. However, the clinically important finding was that the low HL group had significantly lower ADL even in such a situation. Patients in the low HL group have barriers to adhering to medical instructions and accessing preventive services and are subject to delayed diagnosis [11]. Therefore, we suggest that cardiac rehabilitation staff need to intervene given that the low HL group will experience poorer ADL in the future.

### 4.3. Comparison of Analysis Results

Although the FIM scores show a significant difference in the comparative analysis, the low HL group included many patients admitted for heart failure, whereas the high HL group included many patients admitted for myocardial infarction. Heart failure is the final common pathway of several etiologies, such as hypertension and ischemic and valvular heart disease [30]. So, the low HL group might include patients who were on an advanced cardiac disease pathway and were likely to need support with ADL in the future, even if they showed independence at this admission.

We found that low HL can predict ADL impairment from the results of the multiple regression analysis, even after adjusting for other factors, such as MCI. In this study, lifestyle status, such as living together or marriage, were predictors of ADL impairment rather than of a primary diagnosis for admission. In addition, multiple regression analysis identified that living together seemed to harm ADL. However, this reflected the situation that patients with low HL and difficulty in ADL require some support from housemates such as family members.

### 4.4. Clinical Implication

We initially hypothesized that HL and ADL in patients undergoing cardiac rehabilitation would be related, with physical and cognitive function as common factors between them. As a result, HL and ADL showed a significant positive relationship in both the motor and cognitive domains. The low HL group may have impaired ADL, which affects both their physical and cognitive functions. Therefore, cardiac rehabilitation staff need to focus on interventions for patients in the low HL group by considering both their physical and cognitive functions.

The FIM involves assessments for self-care, which includes the knowledge, skill, and motivation domains, and the knowledge domain significantly impacts the ability to provide self-care [31]. Low HL impairs the ability to engage in self-care [31], and the low HL group had significantly low motor FIM scores, which include self-care. Although we could not determine causal relationships in this study, we predict that low HL would result in lower self-care knowledge, which would lead to impairments in self-care ability and ADL. In the clinical setting, self-care is essential for secondary or tertiary prevention of cardiac disease. Therefore, patients in the low HL group would be more likely to have exacerbations of cardiac disease than would patients in the high HL group.

Only 14% of patients with cardiac disease and low HL understand the context of patient education [32]. So, cardiac rehabilitation staff must correctly comprehend the patient’s HL, and patient education suitable for that individual is required. HL screening should be conducted early in hospitalization. Patients with low HL should be given prioritized education, especially considering patients with low HL and MCI, such as by providing pamphlets with content and education that focuses on the family. Furthermore, patients in the low HL group also tend to have impairments in ADL and difficulty in accessing and using health care. Comprehensive support from multiple medical staff, such as proposing and adjusting social services during hospitalization, can be essential for these patients with low HL, in addition to focused ADL practice.

### 4.5. Limitations

The present study has several limitations. First, this study might contain potential biases, such as several cardiac diseases in the primary diagnosis that require different treatments. In addition, the severity of cardiac disease that affects hospitalized activities and the economic status related to HL could not be included in this study. Second, the HLS-14 is a questionnaire assessment and lacks objectivity. Patients with MCI were also included in the analysis, although MCI was adjusted for in the multiple regression analysis. MCI could affect the HLS-14 score due to the HLS-14 being a questionnaire. Third, the FIM showed a ceiling effect; thus, we could not accurately consider whether a direct relationship was present between each item of the FIM and instrumental ADL. Finally, many patients were hospitalized for short-term procedures, such as ablation or CAG, and were excluded from the final analysis, leading to the potential inclusion of selection bias in the study.

## 5. Conclusions

The present multicenter study investigated the relationship between HL and ADL in patients participating in cardiac rehabilitation. Patients in the low HL group had significantly lower ADL than patients in the high HL group. After adjusting for other associated factors in the multiple regression analysis, HLS-14 remained a factor associated with the total FIM score. Our findings underscore the importance of considering HL in cardiac rehabilitation, but effective interventions to improve HL still need to be elucidated. Further study is required to examine interventions to improve HL and ADL, which will lead to a better prognosis for patients with low HL.

## Figures and Tables

**Table 1:** Baseline clinical characteristics of the low and high health literacy groups.

Characteristic	Low HL (*n* = 137)	High HL (*n* = 131)	Z ^†^, χ^2^ Value	*p*-Value
Age (years) *	72.0 [61.0–79.0]	68.0 [58.0–77.0]	1.02 ^†^	0.306
Male ratio (%)	78.1	75.6	0.24	0.623
Body mass index (kg/m^2^) *	22.7 [20.3–26.1]	23.6 [21.1–25.9]	1.33 ^†^	0.183
Education (>13 years) (%)	46.7	55	1.82	0.177
Employment (%)	53.3	58.8	0.82	0.365
Living together (%)	76.6	82.4	1.38	0.240
Smoking (%)			0.01	0.728
No	45.3	40.5		
Past	43.1	46.6		
Current	11.7	13		
Marriage (%)			3.25	0.197
No	19.0	12.2		
Ex	18.2	15.3		
Yes	62.8	72.5		
Admission duration (days) *	16.0 [12.0–22.0]	15.0 [12.0–20.0]	1.27 ^†^	0.203
Discharge home (%)	97.1	98.5	0.59	0.684
Main diagnosis (%)			10.6	0.082
Heart failure	40.9	29.8		
Myocardial infarction	33.6	51.1		
Angina pectoris	11.7	7.6		
Aortic dissection	1.5	0.8		
Aortic aneurysm	0.7	2.3		
Valve diseases	7.3	4.6		
Others	4.4	3.8		
Ejection fraction (%) *	53.0 [38.0–61.3]	52.0 [41.0–62.0]	0.38 ^†^	0.707
Comorbidities (%)				
Hypertension	64.2	61.8	0.17	0.684
Diabetes	35.8	35.1	0.01	0.911
Dyslipidemia	45.3	63.4	8.84	0.003
Congestive heart failure	46.7	38.2	2.00	0.157
Stroke	10.9	9.2	0.24	0.627
Renal dysfunction	27	18.3	2.87	0.090
Peripheral artery disease	8.8	4.6	1.87	0.172
Chronic lung disease	6.6	8.4	0.32	0.569
Mild cognitive impairment	21.9	10.7	6.13	0.013
Charlson Comorbidity Index *	2.0 [1.0–3.0]	1.0 [0.0–3.0]	1.68 ^†^	0.094
Hemoglobin (g/dL) *	13.0 [11.4–14.6]	13.5 [12.0–14.5]	1.49 ^†^	0.137
Creatinine (mg/dL) *	1.0 [0.8–1.3]	0.9 [0.8–1.1]	1.44 ^†^	0.150
eGFR (mL/min/1.73 m^2^)*	55.4 [39.2–69.8]	59.2 [47.1–70.4]	1.68 ^†^	0.093
GNRI *	100.5 [91.5–107.0]	101.9 [92.7–109.2]	0.51 ^†^	0.608
Medications (%)				
Beta blocker (%)	73	75.6	0.23	0.629
ACE-I (%)	25.5	35.1	2.91	0.088
ARB (%)	22.6	29.0	1.43	0.232
Diuretic (%)	55.5	54.2	0.04	0.834
Statin (%)	51.8	73.3	13.1	<0.001
FIM/Motor *	90.0 [88.0–91.0]	91.0 [89.0–91.0]	3.16 ^†^	0.002
FIM/Cognitive *	35.0 [33.0–35.0]	35.0 [35.0–35.0]	3.78 ^†^	<0.001
FIM/Total *	124.0 [120.0–126.0]	125.0 [124.0–126.0]	3.39 ^†^	<0.001
HLS-14/Functional *	19.0 [16.0–22.0]	23.0 [21.0–25.0]	7.46 ^†^	<0.001
HLS-14/Communicative *	13.0 [10.0–15.0]	20.0 [17.0–21.0]	15.3 ^†^	<0.001
HLS-14/Critical *	10.0 [8.0–12.0]	14.0 [12.0–16.0]	8.21 ^†^	<0.001
HLS14/Total *	43.0 [38.0–46.0]	54.0 [52.0–57.5]	23.3 ^†^	<0.001

* Median (interquartile range), ^†^ means Z value. Legends: ACE-I, angiotensin-converting enzyme inhibitor; ARB, angiotensin II receptor blocker; FIM, Functional Independence Measure; eGFR, estimated glomerular filtration rate; GNRI, Geriatric Nutritional Risk Index; HL, health literacy; HLS-14, 14-item Health Literacy Scale.

**Table 2:** Multiple linear regression test for the total score of the Functional Independence Measure.

Characteristic	Univariable Model		Multivariable Model	
	Beta (95% CI)	*p*-Value	Beta (95% CI)	*p*-Value
Age	−0.29 (−0.36 to −0.22)	<0.001	−0.18 (−0.27 to −0.10)	<0.001
Sex	4.87 (2.57 to 7.18)	<0.001	0.98 (−1.24 to 3.20)	0.39
Body mass index	0.32 (0.09 to 0.54)	0.005	−0.11 (−0.37 to 0.15)	0.40
Education	3.12 (1.16 to 5.09)	0.002	−0.21 (−1.97 to 1.55)	0.82
Employment	5.58 (3.67 to 7.48)	<0.001	−0.18 (−2.36 to 2.00)	0.87
Living together	−0.98 (−3.46 to 1.50)	0.44	−3.82 (−6.22 to −1.42)	0.002
Smoking	2.30 (−0.73 to 5.34)	0.14	−0.63 (−3.10 to 1.83)	0.61
Marriage	2.28 (1.50 to 3.06)	<0.001	2.07 (1.22 to 2.91)	<0.001
Heart failure	−5.85 (−7.82 to −3.87)	<0.001	−3.20 (−5.62 to −0.78)	0.010
Myocardial infarction	4.30 (2.34 to 6.26)	<0.001	−0.39 (−2.61 to 1.82)	0.73
Ejection fraction	0.001 (−0.07 to 0.07)	0.97	0.005 (−0.06 to 0.06)	0.88
Stroke	−5.03 (−8.31 to −1.76)	0.003	−2.86 (−5.58 to −0.13)	0.040
Renal disease	−5.00 (−7.31 to −2.68)	<0.001	−0.80 (−2.90 to 1.30)	0.45
MCI	−8.99 (−11.5 to −6.52)	<0.001	−3.98 (−6.44 to −1.51)	0.002
GNRI	0.10 (0.02 to 0.18)	0.012	0.00 (−0.09 to 0.09)	>0.99
HLS-14	0.28 (0.17 to 0.40)	<0.001	0.16 (0.06 to 0.26)	0.002

Legends: CI, confidence interval; GNRI, Geriatric Nutritional Risk Index; HLS-14, 14-item Health Literacy Scale; MCI, mild cognitive impairment.

## Data Availability

The data underlying this article cannot be shared publicly due to privacy concerns of the individuals who participated in the study. The data will be shared upon reasonable request with the corresponding author.
